# Molecular Epidemiology of Human Norovirus Variants from Outbreaks in Zhejiang Province, China, during 2021

**DOI:** 10.1155/2024/7972494

**Published:** 2024-05-30

**Authors:** Yi Sun, Yongjuan Yuan, Haiyan Mao, Lingxuan Su, Qiong Ge, Jian Gao, Changping Xu, Liming Gong

**Affiliations:** ^1^Zhejiang Provincial Center for Disease Control and Prevention, Hangzhou, Zhejiang, China; ^2^Jia Shan Center for Disease Control and Prevention, Jiaxing, Zhejiang, China

## Abstract

**Background:**

Noroviruses are the most frequent cause of epidemic acute viral gastroenteritis in China.

**Objectives:**

The aim of this study was to determine the molecular epidemiological characteristics of norovirus outbreaks and the molecular genetic features of norovirus in Zhejiang Province during 2021.

**Methods:**

First, the local Centers for Disease Control and Prevention in the outbreak area conducted on-site epidemiologic investigations and collected samples from ill patients for initial testing. The general epidemiologic characteristics of the demographic information are presented through descriptive analysis. Positive samples were sent to the Microbiology Laboratory of Zhejiang Provincial Center for Disease Control and Prevention for further verification. The presence of norovirus genogroups I (GI) and II (GII), along with sapovirus, was detected. Subsequently, the specimens positive for norovirus were sequenced for genotyping purposes. Furthermore, the whole genomes of positive samples were sequenced, enabling the characterization of both nucleotide and amino acid differences within the virus. Finally, phylogenetic trees were constructed to further analyze and understand the genetic relationships among the detected viruses.

**Result:**

227 norovirus outbreaks were reported in Zhejiang Province, China, during 2021. Schools were the main setting while January was the peak month for outbreaks. A total of 17 diverse genotypes of norovirus were identified in 2021, and GII.P16-GII.2 was the most frequent genotype (30.19%). Seven genomes (five GI.P4-GI.5 and two GII.P16-GII.2) were obtained. Although GI.P4-GI.5 is considered to be a rare genotype of norovirus, the prevalence might have been underestimated. Capsid microvariation of GII.2 displayed histo-blood group antigen binding patterns compared to the GII.2 prototype, although VP1 sequences were considered to have a minimal impact on antigenicity.

**Conclusion:**

This study revealed the diversity of norovirus strains' genotypes circulating in Zhejiang Province in 2021. Continued molecular surveillance of noroviruses should be strengthened in our further efforts to the development of vaccines.

## 1. Introduction

Norovirus is one of the most important causes of acute gastroenteritis (AGE) of viral etiology globally, especially with successful rotavirus vaccination in some parts of the world [1]. The common symptoms of norovirus infection are diarrhea and vomiting. Norovirus affects people of all ages and has become a serious clinical and economic burden with a median yearly cost of outbreaks reaching $7.6 million in direct medical costs and $165.3 million in productivity losses [2]. It has been estimated that there are approximately 19,496 deaths due to norovirus (95% CI: 8747–38,421) annually [3].

The genus *Norovirus* belongs to the family *Caliciviridae.* Norwalk virus is a single-stranded, positive-stranded RNA virus. The full length of the genome is 7.5 kb∼7.7 kb and contains 3 open reading frames (ORFs): ORF1, ORF2, and ORF3. ORF1 predominantly encodes an RNA-dependent RNA polymerase (RdRp) and other nonstructured proteins. ORF2 codes for primary structure protein (VP1), while ORF3 encodes a minor structural protein (VP2). VP1 is structurally divided into a shell (S) and a protruding (P) domain. The P domain is further divided into P1 and P2 subdomains where the major antigenic site and histo-blood group antigen (HBGA) ligands are located [4, 5]. This species is divided into at least 10 genogroups (GI–GX) and 2 tentative new genogroups (GNA1 and GNA2), which are further subdivided into 49 G-types based on the complete VP1 and 60 P-types based on the partial sequence of the RdRp region [6]. Viruses from genogroups GI, GII, GIV, GVIII, and GIX are known to infect humans, with GII being the most prevalent [7, 8].

Given that only a low infectious dose of norovirus is needed to cause infection, outbreaks can easily occur [9]. Norovirus outbreaks are an important health risk for long-term care facilities such as childcare, schools, and nursing homes, and infections in these settings may lead to an increased risk of hospitalization and death [10, 11]. National norovirus surveillance networks are established by the Chinese Center for Disease Control and Prevention (CDC) monitor in which strains are circulating in humans throughout the year [8, 12]. These networks provide information concerning the epidemiology, pathogenicity, and evolution of the different norovirus genotypes. Based on surveillance data, the objectives of this retrospective study were to describe the molecular epidemiology of norovirus outbreaks and spectrum diversities for different norovirus genotypes in Zhejiang Province, China, during 2021, further illustrating the genetic and phylogenetic characteristics for main norovirus genotypes that year. Understanding these features of noroviruses will be crucial for the development of a long-term strategy to reduce the burden of associated disease and to prevent norovirus outbreaks in a rapidly changing world. Continued surveillance of the changing epidemiologic pattern of noroviruses will better inform the formulation and targeting of candidate vaccines and antivirals. Surveillance network sentinels were set by the Chinese CDC, including the general population, hospitals, day care centers, and long-term care facilities.

## 2. Materials and Methods

The definitions of clusters and outbreaks covered in this study were based on the Technical Guidelines for the Investigation, Prevention, and Control of Norovirus Infection Outbreaks (2015 Edition) published by the Chinese CDC. The local CDC near the sites where the epidemic occurred conducted on-site epidemiologic investigation and collected fecal or swab samples from symptomatic patients for preliminary testing. Positive samples were sent to the Zhejiang Provincial CDC for further inspection, sequencing, and analysis.

Real-time RT-PCR and genotyping were done according to a protocol from the China CDC [12]. Briefly, each specimen was made into a 10% suspension using PBS solution, centrifuged to extract viral nucleic acid from 200 µl supernatant. Norovirus GI and GII types, as well as sapovirus, were detected by the real-time RT-PCR method [12, 13]. GI and GII noroviruses were detected using the Ag-Path kit (Applied Biosystems) with primers (Gog1F, Cog1R, Cog2F, and Cog2R) and TaqMan probes (Ring 1E and Ring 2), while sapovirus was detected with the One Step PrimeScirp™ RT-PCR kit (Takara) according to the manufacturer's instructions [12, 13]. Positive specimens from each outbreak were selected for simultaneous amplification of the polymerase region (RdRp) and capsid region (Capsid), with the amplification primers Mon432 and G1SKR, respectively. Positive PCR products were delivered to Shanghai Biotech Company for sequence determination, and sequences were submitted to the Norovirus Genotyping website (https://www.rivm.nl/mpf7norovirus/typingtool) for genotyping.

A selection of positive samples was analyzed by next generation sequencing (NGS). The NGS procedure was performed, as in a previous study [14]. High-throughput sequencing was performed on Illumina NextSeq 2000 platforms.

To ensure high-quality results, we processed the raw sequencing data using MVP (Microbiome & Virus Analysis Platform) as previously reported [15]. Both genotyping sequences and full-length consensus genomes were analyzed using Geneious Prime 23.0.4 (https://www.geneious.com) software to increase accuracy. A multiple sequence alignment was performed using MUSCLE [16]. Maximum likelihood (ML) analysis was processed with other sequences downloaded from GenBank using RAxML under the GTRGAMMA as the nucleotide substitution model. One thousand bootstrap replicates were run to assess the reliability of the phylogenetic tree [17].

## 3. Results

**Figure 1:**
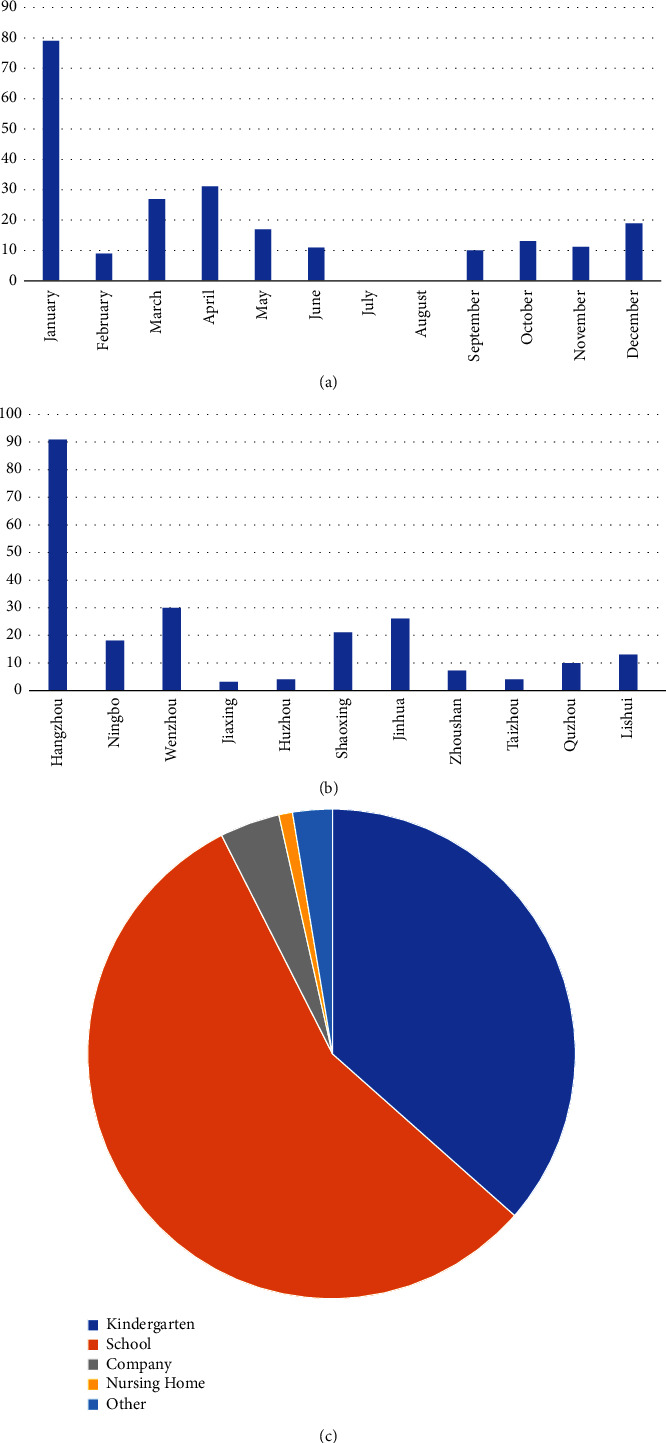
Distribution of norovirus outbreaks during 2021 according to the month, area, and setting. (a) Monthly distribution; (b) area distribution; (c) setting distribution.

**Figure 2:**
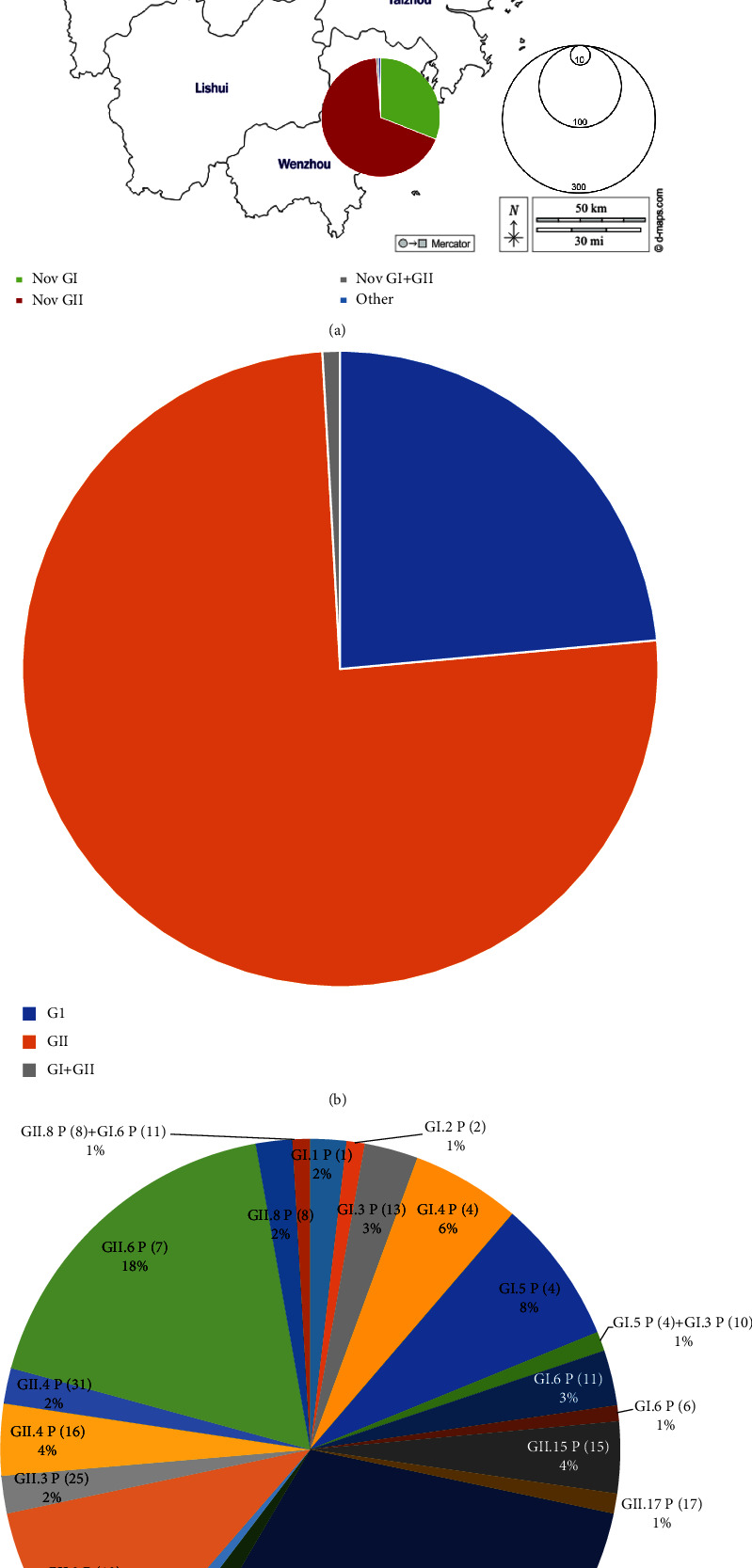
Occupancy chart for different identified noroviruses detected from outbreaks during 2021. (a) Different genotypes showing different city distributions in Zhejiang Province during 2021. Norovirus GI genotypes are indicated in green; norovirus GII genotypes in red; GI mixed infection with GII in grey; others in blue. The size of the circles in different cities represents the size of the sample tested. (b) Percentages for GI, GII, and GI mixed infection with GII. (c) Percentages of different identified noroviruses.

A total of 227 outbreak-associated illnesses were reported in 2021 in Zhejiang Province (Supplementary Table 1). The number of outbreaks peaked in January (79/227), and no cases were reported during July and August (Figure 1(a)). Hangzhou had the highest number of reported outbreaks (91/227), while Jiaxing had the lowest (3/227) (Figure 1(b)). More than half of the outbreaks (55.95%) occurred in schools (127/227; Figure 1(c)). A total of 783 samples were collected from the outbreaks reported in Zhejiang in 2021 (Supplementary Table 2). Of these, 20.43% (160/783) tested positive for norovirus GI, 78.93% (618/783) for norovirus GII, and 0.26% tested positive for sapovirus. All the samples from Huzhou were positive for norovirus GI, while samples from Jiaxing, Zhoushan, Ningbo, and Quzhou were GII. Samples from Shaoxing and Jinhua were a mixture of GI and GII infections. Samples from Hangzhou and Wenzhou were positive not only for GI and GII but also for sapovirus (Figure 2(a)).

Of the 227 reported outbreaks, samples from 114 were received and accounted for 50.22% of the total reported outbreaks. Of the 114 outbreaks with samples received, 106 had a confirmed genotyped norovirus, which accounted for 92.98% of the 114 outbreaks. Among the typed outbreaks, 75.74% (80/106) tested positive for GII, 23.58% (25/106) for GI, and 0.94% (1/106) were mixed GI/GII infections (Figure 2(b)). There were a total of 17 genotypes identified (GI.P1-GI.1, GI.P2-GI.2, GI.P10-GI.3, GI.P13-GI.3, GI.P4-GI.4, GI.P4-GI.5, GI.P6-GI.6, GI.P11-GI.6, GII.P15-GII.15, GII.P17-GII.17, GII.P16-GII.2, GII.P12-GII.3, GII.P25-GII.3, GII.P16-GII.4, GII.P31-GII.4, GII.P7-GII.6, and GII.P8-GII.8). All the GII.4 genotypes detected in this study were the Sydney_2012 variant. There were five outbreaks with mixed infections, including one GI.P4-GI.5/GI.P10-GI.3, two GII.P16-GII.2/GII.P12-GII.3, one GII.P16-GII.2/GII.P16-GII.4, and one GII.P8-GII.8/GI.P11-GI.6 (Figure 2(c)). All the norovirus genotype sequences were deposited in GenBank, with the following accession numbers: OR597872-OR597896 (GI) and OR598618-OR598698 (GII). The Hangzhou area had the highest number of identified genotyped noroviruses (11). The noroviruses genotyped in April were the most diverse, with 13 identified genotypes. GII was the main genotype throughout 2021, especially GII.P16-GII.2, which caused 35 outbreaks across Zhejiang, mainly in schools and kindergartens. GI.P4-GI.5 was the main genotype in norovirus GI but was only detected during February to June 2021. The only mixed infection with GI and GII genotypes was detected from an outbreak in a kindergarten in Hangzhou in June, with the pathogens GI.P11-GI.6/GII.P8-GII.8.

**Figure 3:**
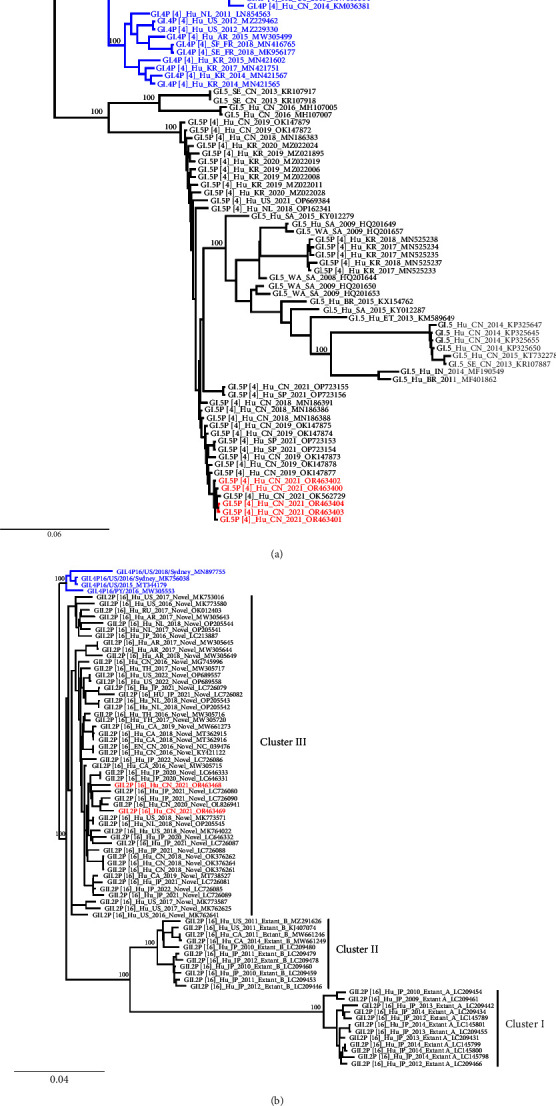
Phylogenetic analysis of noroviruses based on genome sequences. Only 100% bootstrap values are shown. Sequences from this study are highlighted in red. (a) The phylogenetic tree for GI.P4-GI.5; GI.P4-GI.4 from GenBank was used as the root. Sequences of norovirus GI.P4-GI.4 are indicated in blue and those of GI.P4-GI.5 in black. (b) The phylogenetic tree for GII.P16-GII.2; GII.P16-GII.4 from GenBank was used as the root. Sequences of norovirus GII.P16-GII.4 are indicated in blue and those of GII.P16-GII.2 in black.

Seven norovirus genomes were obtained in this study, including five GI.P4-GI.5 and two GII.P16-GII.2. Comparisons of the genomes showed that the five noroviruses identified as GI.P4-GI.5 shared 99.8% and 99.7% identity at the nucleotide (nt) and amino acid (aa) level of the coding region, respectively. A similar analysis with the GII.P16-GII.2 indicated a 96.8% and 93.9% identity at the nt and aa level of the coding region, respectively. All the genome sequences were deposited in GenBank, with accession numbers: OR463400–OR463404 (GI.P4-GI.5) and OR463468–OR463469 (GII.P16-GII.2). We aligned two GII.2 capsid sequences and compared the sequences to the prototype GII.2 strain, GII.2 1976 Snow Mountain Virus (SMV), and other GII.2 strains circulated during the last few years [13]. Our two GII.2 strains (ZJ_3_2021 and ZJ_5_2021) had 97.7% amino acid similarity to GII.2 1976 SMV. Ile and were detected in these two GII.2 (ZJ_3_2021 and ZJ_5_2021) at the 256^th^ amino acid position in the VP1 region, while the HBGA binding site remained conserved compared to SMV from 1976 (Table 1). We downloaded all the GI.P4-GI.5 genomes, several GI.5 partial sequences, and GI.P4-GI.4 from GenBank and constructed a phylogenetic tree with the GI.P4-GI.5 identified in this study. The tree showed that our five GI.P4-GI.5 strains clustered together into a monophyletic clade, with the closest phylogenetic relationship with a genome isolated from Guangzhou during 2021 (Figure 3(a)). Additionally, we built a phylogenetic tree using the GII.P16-GII.2 identified in this study and other GII.P16-GII.2 sequences randomly downloaded from the GenBank database. Several GII.P16-GII.4 Sydney_2012 sequences downloaded from GenBank were used as outgroups. The two GII.P16-GII.2 genomes obtained in this study were assigned to cluster III with the novel GII.P16-GII.2 downloaded from the database. Cluster III was compared to clusters I and II, which were composed of extant A and B subclades of GII.P16-GII.2, and mainly from Japan (Figure 3(b)). These two GII.P16-GII.2 sequences showed close phylogenetic relationships with sequences from China and Japan isolated during 2020 to 2021 (Figure 3(b); LC726080, LC726090, and OL826941).

## 4. Discussion

Due in part to the public health impact, the epidemiology and diversity of noroviruses have been investigated worldwide [18, 19]. Our results demonstrated that a relatively large percentage of infections caused by norovirus occur in the winter months, which was consistent with other reports [12, 20, 21]. Almost all outbreaks in our study occurred in kindergartens and schools, which have likewise been identified in other provinces in China and other Asian countries [21]. Jin et al. believed that the high proportion of norovirus outbreaks in childcare centers and schools in China was different from that in Western countries because of the high population density in these settings and the enhanced monitoring and reporting of any outbreaks in schools in China [12]. However, we demonstrated that COVID-19 measures implemented in 2021 may have affected the epidemiological pattern of norovirus; similar circumstances were reported in which control measures, such as quarantine at home, keeping a social distance, wearing face masks, and frequent hand-washing, may help reduce the risk of norovirus infection, as with other pathogens [14, 22]. Moreover, we observed high variability in reporting rates among cities because 40.08% of reported norovirus outbreaks in Zhejiang during the study period occurred in Hangzhou, the capital city of Zhejiang Province, while other cities had only a few outbreaks reported. Additionally, surveillance data showed that the most genotyped norovirus and a sapovirus were identified as causing outbreaks in the Hangzhou area. This is likely due to difficulties reporting in some divisions because of insufficient resources, such as understaffing caused by COVID-19 measures [23]. However, rather than the influence of COVID-19, similar phenomena were also explained in other studies by understaffing at health departments or the differences in state-mandated reporting criteria for healthcare-associated outbreaks in the United States [24].

Worldwide, noroviruses have been responsible for most viral-associated AGE for nearly three decades because of the chronologic sequential emergence of novel variants every 2 to 4 years. Recombination of human noroviruses is frequently observed and is thought to be an important mechanism by which genetic diversity is generated. A previous study showed that GI.P4-GI.5 was identified as an intergenotype recombinant of GI.P4-GI.4 and GI.P5-GI.5 [25]. It was first detected in India in 2016, and there are now a total of 32 partial or complete GI.P4-GI.5 sequences available in GenBank. This norovirus genotype, GI.P4-GI.5, caused a peak in outbreaks in the first 6 months of 2021, might be considered as a rarely reported norovirus genotype according to previous studies [3, 12, 25]. However, we believe that the prevalence of GI.P4-GI.5 might have been underestimated. The support for this hypothesis is based on our inference that the lineage sorting process of GI.P4-GI.5 in different areas might be ongoing with genetic diversity gradually evolving. We found that it has segregated into several distinct genetic subclades mainly from China, South Korea, Spain, Netherlands, South Africa, and the United States. This genotype has circulated, quickly spread, and evolved endemically on multiple continents after 2016. In addition, GI.5 might be easy to detect in water sample sources, such as sewage. Water might be an important storage pool for norovirus [26]. The phylogenetic tree demonstrated GI.5 circulation and transmission among populations and the environment in countries. Many cities in China have reported the GI.P4-GI.5 inspection [27]. More detailed transmission and epidemiological studies on this genotype need to be conducted in the future.

Compared to the GI.P4-GI.5 genotype, GII.P16-GII.2 is considered to be a more prevalent norovirus genotype [28]. VP1 sequences of GII.2 viruses have been evolving linearly for decades, which were considered to have a minimal impact on the antigenicity of GII.2 viruses with completed lineage sorting in the evolutionary pattern of VP1 [29]. Compared to GII.2 1976 SMV, our GII.2 displayed a reduced affinity to A saliva HBGA binding patterns according to the study from Mallory et al. in 2020 [5]. A mutation occurred at the 256^th^ amino acid position in the VP1 region proximal to the HBGA SiteI where the amino acid changed from Val to Ile in sequences dating from 2016 and beyond. Furthermore, the amino acids within the HBGA binding site of the newer GII.2 variants emerging after 2016 remained unchanged in agreement with previous research outcomes [30]. In this study, the genotypes that caused the most outbreaks belonged to GII, especially GII.P16-GII.2 from our observations. GII.P16-GII.2 was mainly associated with outbreaks in kindergartens and schools, consistent with a study in Japan [31]. GII.P16-GII.2 norovirus was originally detected in outbreaks from 2009 to 2010 in Osaka, Japan, before emerging in China in 2016, then becoming predominant in outbreaks in other Asian countries [32]. Since 2015, multiple other genotypes harboring a nearly identical GII.P16 polymerase have been identified, including GII.1, GII.2, GII.3, GII.10, and GII.12. Barclay et al. reported that viruses harboring the GII.P16 polymerase accounted for 43.1% of outbreaks with three phylogenetic clades separated as “extant A,” “extant B,” and “novel” [32]. Our GII.P16-GII.2 was in cluster III, with other “novel” strains that diverged from a common ancestor with both clusters I (extant A) and II (extant B), with a close phylogenetic relationship with sequences from Changsha, China, and Tokyo, Japan, during 2020 2021. GII.P16-GII.2, with GII.P4-GII.4 Sydney_2012, was considered as a strong pandemic replication competent strain that cocirculates and may cause severe gastroenteritis and lead to poor clinical outcomes [33]. Therefore, it is necessary to understand the evolutionary patterns of the virus and variants in major antigenic sites in genotypes with different clinical symptoms in a corollary study.

There were several limitations to our study. First, owing to the impact of COVID-19, the number of norovirus cases and specimens was limited, especially for the Lishui and Taizhou areas. Second, detailed analysis of the relationship between the genotypes identified in this study and the clinical course of disease needs to be addressed in future research. Third, although GII.4 was not the main genotype detected in this study, evolution of GII.4 strains and the periodic emergence of the distinct variants have dominated the global landscape for over two decades. Other genotypes, such as GII.2, have recently posed a challenge to the dominance of GII.4 in various geographic regions and time frames. Therefore, a comprehensive study on the GII.4 genotype is warranted in our future research.

## 5. Conclusion

In this study, we shed light on novel information by revealing new molecular epidemiological information and diversity of microbiology spectrum data on outbreaks of norovirus in Zhejiang Province during 2021. Additionally, we reported the genetic and phylogenetic characteristics of two predominant norovirus genotypes, GI.P4-GI.5 and GII.P16-GII.2, circulating in the local area, which improved our understanding of norovirus transmission and viral evolution. Continued surveillance and comprehensive comparison of the molecular epidemiological characteristics and genetic features of norovirus before, during, and after the pandemic of COVID-19 are required to increase understanding of causal-effect relationship between predominant strains and the pattern of outbreaks, which may aid norovirus control and prevention and reduce the overall burden of norovirus disease.

## Figures and Tables

**Table 1:** Sequence variation between 2021 GII.2 outbreak strains used in this study and prototype strain GII.2 Snow Mountain Virus (SMV) 1976 and other GII.2 sequences circulated in the last few years.

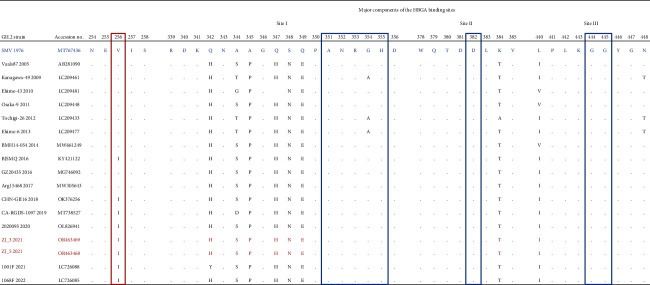

The blue sequence is the prototype strain SMV1976; the red two sequences are the two GII.2 sequences ZJ_3_2021 and ZJ_5_2021 detected in this study; the other black sequences are the VP1 sequences of GII.2 from 2005 to 2022 obtained from the GenBank database. The three blue rectangular boxes represent the three sites where the virus binds to HBGA, Site I, Site II, and Site III; “.” indicates that the amino acid at the corresponding position is identical to that of the reference strain, SMV1976.

## Data Availability

All the norovirus genotype sequences and genome sequences were deposited in GenBank, with the following accession numbers: OR597872-OR597896 (GI), OR598618-OR598698 (GII), OR463400–OR463404 (GI.P4-GI.5), and OR463468–OR463469 (GII.P16-GII.2).

## Supplementary Materials

Supplementary Materials Supplementary Table 1: information on each reported norovirus outbreak during 2021 in Zhejiang Province. Note: “N/A” in “Genotype” column represents the unsuccessful norovirus genotype result. Supplementary Table 2: information on samples collected from the outbreaks reported in Zhejiang in 2021.
