# Supplementary material for: Molecular Epidemiology of Human Norovirus Variants from Outbreaks in Zhejiang Province, China, during 2021
Source: Adv Virol. 2024 May 30;2024:7972494. doi: 10.1155/2024/7972494 (PMC11156503; doi:10.1155/2024/7972494)
Supplement: Supplementary Materials — Supplementary Table 1: information on each reported norovirus outbreak during 2021 in Zhejiang Province. Note: “N/A” in “Genotype” column represents the unsuccessful norovirus genotype result. Supplementary Table 2: information on samples collected from the outbreaks reported in Zhejiang in 2021. [file 7972494.f1.docx]

Supplementary Table1 Information on each reported norovirus outbreak during 2021 in Zhejiang Province. Note: “N/A” in “Genotype” column represent the unsuccessful norovirus genotype result.

| Time | Area | Genotype | Settings |
| --- | --- | --- | --- |
| 2021/1/1 | Hangzhou | N/A | School |
| 2021/1/1 | Zhoushan | N/A | School |
| 2021/1/1 | Hangzhou | N/A | School |
| 2021/1/1 | Ningbo | GII.3P[12] | Kindergarten |
| 2021/1/2 | Hangzhou | N/A | School |
| 2021/1/4 | Zhoushan | N/A | Kindergarten |
| 2021/1/4 | Wenzhou | N/A | School |
| 2021/1/6 | Quzhou | GII.2P[16] | School |
| 2021/1/6 | Jinhua | N/A | School |
| 2021/1/7 | Quzhou | N/A | School |
| 2021/1/7 | Shaoxing | GII.6P[7] | Kindergarten |
| 2021/1/7 | Wenzhou | GII.2P[16] | School |
| 2021/1/7 | Wenzhou | GII.2P[16] | Kindergarten |
| 2021/1/7 | Quzhou | GII.4P[16] | School |
| 2021/1/8 | Hangzhou | N/A | Kindergarten |
| 2021/1/8 | Ningbo | GII.3P[12] | Kindergarten |
| 2021/1/8 | Hangzhou | N/A | Kindergarten |
| 2021/1/8 | Hangzhou | N/A | School |
| 2021/1/8 | Wenzhou | N/A | School |
| 2021/1/8 | Wenzhou | GII.2P[16] | School |
| 2021/1/8 | Shaoxing | N/A | School |
| 2021/1/8 | Wenzhou | GII.3P[12] | School |
| 2021/1/9 | Hangzhou | GII.2P[16] | Kindergarten |
| 2021/1/10 | Hangzhou | N/A | School |
| 2021/1/12 | Hangzhou | N/A | School |
| 2021/1/12 | Hangzhou | N/A | School |
| 2021/1/12 | Jinhua | GII.2P[16] | School |
| 2021/1/13 | Quzhou | N/A | Company |
| 2021/1/13 | Jinhua | GII.6P[7] | Kindergarten |
| 2021/1/14 | Shaoxing | GII.6P[7] | School |
| 2021/1/14 | Wenzhou | GII.2P[16] | Other |
| 2021/1/14 | Wenzhou | GII.4P[31] | Kindergarten |
| 2021/1/14 | Hangzhou | N/A | School |
| 2021/1/14 | Hangzhou | GII.2P[16] | School |
| 2021/1/14 | Ningbo | GII.2P[16] | School |
| 2021/1/14 | Hangzhou | N/A | School |
| 2021/1/14 | Quzhou | GII.2P[16] | School |
| 2021/1/14 | Quzhou | GII.2P[16] | School |
| 2021/1/14 | Ningbo | GII.2P[16]+GII.3P[12] | School |
| 2021/1/15 | Wenzhou | N/A | Kindergarten |
| 2021/1/15 | Wenzhou | N/A | Kindergarten |
| 2021/1/15 | Hangzhou | N/A | School |
| 2021/1/15 | Hangzhou | GII.2P[16] | School |
| 2021/1/15 | Hangzhou | GII.3P[12] | Kindergarten |
| 2021/1/15 | Hangzhou | GII.2P[16] | Kindergarten |
| 2021/1/15 | Hangzhou | N/A | School |
| 2021/1/16 | Hangzhou | GII.2P[16] | School |
| 2021/1/16 | Hangzhou | N/A | School |
| 2021/1/16 | Hangzhou | N/A | School |
| 2021/1/16 | Hangzhou | N/A | Kindergarten |
| 2021/1/17 | Hangzhou | N/A | School |
| 2021/1/18 | Ningbo | N/A | Nursing Home |
| 2021/1/18 | Hangzhou | GII.3P[12] | Kindergarten |
| 2021/1/20 | Zhoushan | N/A | School |
| 2021/1/20 | Ningbo | N/A | School |
| 2021/1/20 | Hangzhou | N/A | Company |
| 2021/1/20 | Hangzhou | N/A | School |
| 2021/1/20 | Hangzhou | N/A | Kindergarten |
| 2021/1/21 | Hangzhou | GII.3P[12] | Company |
| 2021/1/21 | Ningbo | N/A | School |
| 2021/1/21 | Lishui | N/A | School |
| 2021/1/21 | Hangzhou | GII.2P[16] | Kindergarten |
| 2021/1/21 | Jinhua | GII.2P[16] | School |
| 2021/1/21 | Lishui | N/A | School |
| 2021/1/21 | Quzhou | GII.2P[16] | School |
| 2021/1/21 | Zhoushan | N/A | Kindergarten |
| 2021/1/21 | Hangzhou | GII.2P[16] | School |
| 2021/1/22 | Hangzhou | GII.2P[16] | School |
| 2021/1/22 | Wenzhou | N/A | School |
| 2021/1/22 | Shaoxing | GII.2P[16] | School |
| 2021/1/22 | Hangzhou | GII.2P[16]+GII.3P[12] | Kindergarten |
| 2021/1/23 | Hangzhou | N/A | School |
| 2021/1/23 | Hangzhou | N/A | Kindergarten |
| 2021/1/23 | Hangzhou | GII.3P[12] | Kindergarten |
| 2021/1/23 | Hangzhou | GII.3P[12] | Kindergarten |
| 2021/1/26 | Hangzhou | GII.2P[16] | School |
| 2021/1/26 | Wenzhou | GII.3P[25] | School |
| 2021/1/27 | Lishui | N/A | School |
| 2021/1/28 | Jinhua | N/A | School |
| 2021/2/1 | Hangzhou | GII.4P[31] | Nursing Home |
| 2021/2/2 | Hangzhou | N/A | Kindergarten |
| 2021/2/5 | Hangzhou | N/A | Kindergarten |
| 2021/2/7 | Hangzhou | GII.3P[12] | Company |
| 2021/2/18 | Hangzhou | GI.5P[4] | Company |
| 2021/2/24 | Wenzhou | GII.2P[16] | Other |
| 2021/2/25 | Hangzhou | GII.2P[16] | Company |
| 2021/2/26 | Wenzhou | N/A | School |
| 2021/2/28 | Hangzhou | N/A | School |
| 2021/3/2 | Shaoxing | N/A | School |
| 2021/3/2 | Lishui | N/A | Kindergarten |
| 2021/3/4 | Ningbo | GII.2P[16]+GII.4P[16] | School |
| 2021/3/4 | Jinhua | N/A | School |
| 2021/3/4 | Zhoushan | N/A | Kindergarten |
| 2021/3/5 | Hangzhou | GII.2P[16] | School |
| 2021/3/5 | Hangzhou | GII.2P[16] | Kindergarten |
| 2021/3/6 | Ningbo | N/A | School |
| 2021/3/6 | Quzhou | GII.4P[16] | Kindergarten |
| 2021/3/7 | Hangzhou | GI.5P[4] | Company |
| 2021/3/11 | Jiaxing | GII.2P[16] | Kindergarten |
| 2021/3/11 | Jinhua | GII.6P[7] | School |
| 2021/3/13 | Ningbo | GII.3P[12] | Kindergarten |
| 2021/3/13 | Ningbo | N/A | Kindergarten |
| 2021/3/15 | Taizhou | N/A | Kindergarten |
| 2021/3/17 | Jinhua | GI.3P[13] | School |
| 2021/3/18 | Wenzhou | N/A | Kindergarten |
| 2021/3/18 | Hangzhou | N/A | School |
| 2021/3/19 | Zhoushan | N/A | Kindergarten |
| 2021/3/22 | Jinhua | GI.5P[4] | School |
| 2021/3/23 | Hangzhou | GII.8P[8] | School |
| 2021/3/24 | Hangzhou | GII.2P[16] | School |
| 2021/3/25 | Shaoxing | GII.2P[16] | Other |
| 2021/3/25 | Wenzhou | N/A | School |
| 2021/3/26 | Taizhou | N/A | School |
| 2021/3/26 | Zhoushan | N/A | Kindergarten |
| 2021/3/31 | Wenzhou | N/A | School |
| 2021/4/1 | Lishui | N/A | Kindergarten |
| 2021/4/1 | Wenzhou | N/A | School |
| 2021/4/8 | Taizhou | N/A | School |
| 2021/4/8 | Jiaxing | GII.4P[16] | School |
| 2021/4/9 | Hangzhou | N/A | Kindergarten |
| 2021/4/9 | Shaoxing | N/A | School |
| 2021/4/9 | Hangzhou | GI.2P[2] | Kindergarten |
| 2021/4/9 | Hangzhou | N/A | Kindergarten |
| 2021/4/14 | Hangzhou | GI.3P[13] | School |
| 2021/4/14 | Hangzhou | N/A | School |
| 2021/4/14 | Jinhua | GI.6P[11] | School |
| 2021/4/15 | Shaoxing | GII.17P[17] | Other |
| 2021/4/15 | Taizhou | N/A | School |
| 2021/4/15 | Huzhou | N/A | Kindergarten |
| 2021/4/16 | Hangzhou | N/A | Kindergarten |
| 2021/4/16 | Jinhua | GII.15P[15] | School |
| 2021/4/17 | Ningbo | GII.6P[7] | School |
| 2021/4/21 | Hangzhou | GI.5P[4] | School |
| 2021/4/22 | Hangzhou | GII.2P[16] | School |
| 2021/4/22 | Shaoxing | N/A | Kindergarten |
| 2021/4/22 | Hangzhou | GII.3P[12] | Kindergarten |
| 2021/4/22 | Wenzhou | N/A | School |
| 2021/4/23 | Jiaxing | GI.6P[6] | School |
| 2021/4/23 | Wenzhou | GI.4P[4] | School |
| 2021/4/24 | Hangzhou | GI.5P[4] | School |
| 2021/4/24 | Ningbo | N/A | Kindergarten |
| 2021/4/24 | Lishui | N/A | School |
| 2021/4/24 | Wenzhou | GI.4P[4] | Kindergarten |
| 2021/4/27 | Wenzhou | GI.4P[4] | School |
| 2021/4/28 | Wenzhou | GII.3P[25] | Other |
| 2021/4/29 | Wenzhou | N/A | School |
| 2021/5/2 | Jinhua | GII.6P[7] | Kindergarten |
| 2021/5/8 | Huzhou | N/A | School |
| 2021/5/8 | Jinhua | GI.5P[4]+GI.3P[10] | School |
| 2021/5/10 | Wenzhou | GI.4P[4] | School |
| 2021/5/14 | Wenzhou | GI.4P[4] | School |
| 2021/5/17 | Hangzhou | GI.3P[13] | Kindergarten |
| 2021/5/17 | Wenzhou | N/A | Other |
| 2021/5/18 | Hangzhou | N/A | Kindergarten |
| 2021/5/20 | Jinhua | GI.5P[4] | School |
| 2021/5/21 | Hangzhou | GI.1P[1] | Company |
| 2021/5/21 | Wenzhou | GII.2P[16] | Kindergarten |
| 2021/5/21 | Hangzhou | N/A | School |
| 2021/5/27 | Quzhou | GI.5P[4] | School |
| 2021/5/28 | Shaoxing | N/A | School |
| 2021/5/28 | Hangzhou | GI.6P[11] | Kindergarten |
| 2021/5/31 | Shaoxing | GI.6P[11] | School |
| 2021/5/31 | Ningbo | N/A | School |
| 2021/6/3 | Jinhua | GI.1P[1] | School |
| 2021/6/3 | Wenzhou | GI.4P[4] | School |
| 2021/6/3 | Hangzhou | N/A | Kindergarten |
| 2021/6/4 | Shaoxing | N/A | School |
| 2021/6/7 | Hangzhou | GII.15P[15] | School |
| 2021/6/8 | Hangzhou | N/A | School |
| 2021/6/10 | Lishui | N/A | School |
| 2021/6/11 | Shaoxing | GI.5P[4] | School |
| 2021/6/11 | Hangzhou | GII.8P[8]+GI.6P[11] | Kindergarten |
| 2021/6/23 | Jinhua | GII.15P[15] | School |
| 2021/6/25 | Shaoxing | N/A | School |
| 2021/9/3 | Hangzhou | GII.6P[7] | Kindergarten |
| 2021/9/9 | Hangzhou | GII.6P[7] | Kindergarten |
| 2021/9/10 | Jinhua | GII.8P[8] | Company |
| 2021/9/12 | Hangzhou | GII.6P[7] | School |
| 2021/9/16 | Hangzhou | GII.6P[7] | Kindergarten |
| 2021/9/27 | Jinhua | GII.6P[7] | Kindergarten |
| 2021/9/28 | Jinhua | N/A | Kindergarten |
| 2021/9/29 | Hangzhou | N/A | School |
| 2021/9/30 | Hangzhou | N/A | Kindergarten |
| 2021/9/30 | Lishui | N/A | Kindergarten |
| 2021/10/11 | Jinhua | GII.6P[7] | School |
| 2021/10/11 | Jinhua | GII.6P[7] | Kindergarten |
| 2021/10/14 | Lishui | N/A | Kindergarten |
| 2021/10/14 | Jinhua | GII.6P[7] | School |
| 2021/10/14 | Hangzhou | GII.6P[7] | Kindergarten |
| 2021/10/15 | Hangzhou | N/A | School |
| 2021/10/15 | Jinhua | GII.6P[7] | Kindergarten |
| 2021/10/20 | Lishui | N/A | Kindergarten |
| 2021/10/20 | Hangzhou | GII.2P[16] | School |
| 2021/10/22 | Hangzhou | N/A | Kindergarten |
| 2021/10/22 | Shaoxing | N/A | School |
| 2021/10/28 | Hangzhou | N/A | Kindergarten |
| 2021/10/29 | Hangzhou | N/A | School |
| 2021/11/3 | Shaoxing | N/A | School |
| 2021/11/5 | Shaoxing | N/A | Kindergarten |
| 2021/11/6 | Hangzhou | N/A | Kindergarten |
| 2021/11/12 | Huzhou | N/A | School |
| 2021/11/15 | Hangzhou | N/A | Kindergarten |
| 2021/11/15 | Hangzhou | N/A | Kindergarten |
| 2021/11/20 | Ningbo | N/A | School |
| 2021/11/22 | Ningbo | N/A | Kindergarten |
| 2021/11/25 | Jinhua | GII.6P[7] | Kindergarten |
| 2021/11/26 | Jinhua | GII.6P[7] | Kindergarten |
| 2021/11/28 | Ningbo | GII.4P[16] | School |
| 2021/12/2 | Hangzhou | N/A | Kindergarten |
| 2021/12/2 | Lishui | N/A | Kindergarten |
| 2021/12/3 | Shaoxing | N/A | Kindergarten |
| 2021/12/3 | Hangzhou | N/A | Kindergarten |
| 2021/12/3 | Shaoxing | N/A | Kindergarten |
| 2021/12/3 | Hangzhou | N/A | Kindergarten |
| 2021/12/3 | Shaoxing | N/A | School |
| 2021/12/5 | Hangzhou | N/A | School |
| 2021/12/6 | Wenzhou | GII.2P[16] | School |
| 2021/12/9 | Quzhou | N/A | School |
| 2021/12/10 | Hangzhou | N/A | School |
| 2021/12/10 | Huzhou | N/A | School |
| 2021/12/12 | Lishui | N/A | School |
| 2021/12/18 | Ningbo | GII.6P[7] | Kindergarten |
| 2021/12/20 | Shaoxing | GII.15P[15] | School |
| 2021/12/23 | Hangzhou | N/A | Kindergarten |
| 2021/12/29 | Lishui | N/A | School |
| 2021/12/29 | Jinhua | N/A | School |
| 2021/12/30 | Hangzhou | N/A | Kindergarten |

Note: “N/A” in “Genotype” column represent the unsuccessful norovirus genotype result.

Supplementary Table2 Information on sample collected from the outbreaks reported in Zhejiang in 2021.

| Area | Sample | Norovirus | | | Others |
| --- | --- | --- | --- | --- | --- |
|  |  | GI | GII | GI mixed with GII | Sapovirus |
| Hangzhou | 272 | 61 | 208 | 2 | 1 |
| Jiaxing | 13 | 0 | 13 | 0 | 0 |
| Ningbo | 66 | 0 | 66 | 0 | 0 |
| Zhoushan | 40 | 0 | 40 | 0 | 0 |
| Wenzhou | 171 | 53 | 116 | 1 | 1 |
| Huzhou | 7 | 7 | 0 | 0 | 0 |
| Quzhou | 21 | 0 | 21 | 0 | 0 |
| Shaoxing | 50 | 3 | 47 | 0 | 0 |
| Jinhua | 143 | 36 | 107 | 0 | 0 |
|  | 783 | 160 | 618 | 3 | 2 |
